# Interpersonal psychotherapy versus treatment as usual for PTSD and depression among Sichuan earthquake survivors: a randomized clinical trial

**DOI:** 10.1186/1752-1505-8-14

**Published:** 2014-09-04

**Authors:** Rui Fang Jiang, Hui Qi Tong, Kevin L Delucchi, Thomas C Neylan, Qijia Shi, Susan M Meffert

**Affiliations:** 1 Wuhan Hospital for Psychotherapy, Wuhan Mental Health Center, Tongji Medical College Huazhong University of Science and Technology, Kaiming Road 44#, Wuhan 430019, China; 2 Department of Psychiatry, University of California, San Francisco, California, USA

**Keywords:** Earthquake, Disaster, Mental health, Posttraumatic stress disorder, Depression, Domestic violence

## Abstract

**Background:**

Without effective treatment, PTSD and depression can cause persistent disability in disaster-affected populations.

**Methods:**

Our objective was to test the efficacy of Interpersonal Psychotherapy (IPT) delivered by trained local personnel compared with treatment as usual (TAU) for Posttraumatic Stress Disorder (PTSD) and Major Depressive Disorder (MDD) among adults affected by the Sichuan 2008 earthquake. A small randomized controlled trial of IPT + TAU versus TAU alone was delivered by local mental health personnel in Shifang, China. Between July 2011 and January 2012, 49 adults ≥ 18 years with PTSD, MDD or both were enrolled and randomized to 12 weekly sessions of IPT + TAU (27) or TAU (22) alone x 12 weeks. IPT was then offered to the TAU group. Unblinded follow up assessments were conducted at three and six months. IPT was a 12 session, weekly one hour treatment delivered by local personnel who were trained and supervised in IPT. TAU was continuation of prescribed psychotropic medication (if applicable) and crisis counseling, as needed.

Main Outcome(s) and Measures (s): Clinician Administered PTSD Scale (CAPS) PTSD diagnosis; Structured Clinical Interview for DSM-IV (SCID) for MDD diagnosis. Secondary measures included PTSD/depression symptoms, interpersonal conflict/anger, social support, self-efficacy and functioning.

**Results:**

Using an intent-to-treat analysis, 22 IPT + TAU and 19 TAU participants were compared at three months post-baseline. A significantly greater reduction of PTSD and MDD diagnoses was found in the IPT group (51.9%, 30.1%, respectively) versus the TAU group (3.4%, 3.4%, respectively). Despite the small sample, the estimates for time-by-condition analyses of target outcomes (2.37 for PTSD (p = .018) and 1.91 for MDD (p = .056)) indicate the improvement was better in the IPT + TAU condition versus the TAU group. Treatment gains were maintained at 6 months for the IPT group. A similar treatment response was observed in the TAU group upon receipt of IPT.

**Conclusions:**

This initial study shows that IPT is a promising treatment for reducing PTSD and depression, the two major mental health disorders affecting populations surviving natural disaster, using a design that builds local mental health care capacity.

**Trial Registration:**

ClinicalTrials.Gov number, NCT01624935.

## Background and Rationale

Seventy-five percent of people living with serious mental illness in Low and Middle Income Countries (LMICs) never receive treatment [1]. When natural disaster occurs in LMICs, additional mental health care needs emerge. The 2008 Sichuan earthquake resulted in the deaths of 69,179 people, including more than 5,000 students in school at the time of the event. PTSD and depression were estimated to affect 65% of the surviving population [2-6].

We conducted a mental health care needs assessment (2009–2010) in collaboration with the Wuhan Hospital for Psychotherapy (WHP), an early mental health care responder, that included interviews and focus groups with survivors, healthcare providers and community leaders. We found that depression, PTSD and relationship distress were significant problems and were commonly ascribed to interpersonal loss, spousal conflict after children’s deaths, and transitions related to material and job loss. Individual treatment (versus group) was desired. Although psychotherapies such as exposure therapy and cognitive processing therapy are first line treatments for trauma-related disorders in Europe and North America, and have been used successfully in LMICs [7], our mental health care needs assessment in this disaster-affected Sichuan population indicated that PTSD symptoms were one component of broader emotional distress and pervasive disruption of relationships. Drawing on prior studies showing the efficacy of IPT in LMICs and with trauma-affected populations [8-11], we theorized that the relational focus of Interpersonal Psychotherapy (IPT) would be well-aligned with local needs. IPT delivered by area personnel was selected in order to contribute to capacity building.

IPT is a 12-week structured psychotherapy developed by Klerman and Weissman in the 1980s and an established first line treatment for depression in the U.S. [12,13]. The goal of IPT is to examine and change current relationships and social support in order to improve mood and anxiety symptoms. IPT has been modified for other uses, including delivery by trained paraprofessionals in culturally distinct settings [8]. To our knowledge, this is the first study of IPT for disaster-related mental disorders in LMICs and the first to use eligibility criteria that allow for PTSD and/or Major Depressive Disorder (MDD)—two major mental health sequelae of traumatic stress [14].

### Objectives and Hypotheses

This article reports the results of a pilot randomized controlled clinical trial of IPT plus treatment as usual (TAU) versus TAU alone for Sichuan earthquake survivors, using a capacity building design with permissive eligibility criteria. The study took place between July 2011 and April 2012. Our purpose was to pilot test the efficacy of IPT for PTSD and MDD (primary outcomes), interpersonal factors and functioning. We hypothesized that IPT + TAU would reduce diagnosis and symptoms of PTSD and MDD, reduce interpersonal conflict and improve social support and functioning, relative to TAU, alone.

## Methods

### Participants

Participants were 18 years of age or older, able to attend weekly sessions, give verbal informed consent, met criteria for PTSD as assessed by the CAPS and/or MDD, as assessed by the SCID. Exclusion criteria were psychosis, cognitive dysfunction or substance abuse/dependence requiring a different type of care. Participants taking prescribed psychotropic medication, including Selective Serotonin Reuptake Inhibitors (SSRIs), Selective Serotonin and Norepinephrine Reuptake Inhibitors (SNRIs) and benzodiazepines, were eligible for the study.

Informed by our prior mental health care needs assessment, recruitment efforts focused on locations with high concentrations of individuals who continued to suffer mental health effects from the 2008 earthquake despite the intervening time. Two of these were mental health care centers (Shifang Counseling Center and Shifang People’s Hospital) and the third was a high school that had been heavily affected by the earthquake. Participants were referred to the study by mental health care providers and recruited from the high school through a general screening. No siblings, parents or teachers of study participants were included. Prospective participants provided informed consent prior to screening for study eligibility.

### Procedures

This was a two-group parallel, randomized trial of IPT + TAU versus TAU in Sichuan, China. Following the 12 week IPT + TAU, those assigned to TAU were offered IPT treatment. All study procedures were approved by the University of California San Francisco Committee on Human Research and its counterpart at WHP. Verbal consent was used because of incomplete literacy.

Given that this was a small pilot study of IPT for disaster survivors, sample size was not based on hypothesis testing but on issues of feasibility within budget constraints. We estimated that at least 20 individuals per arm would be needed to detect a clinically relevant treatment effect [15,16]. Individuals were randomized to IPT + TAU or TAU in a 1:1 fashion, stratified by gender using blocks of size six to ensure approximate balance.

Seven mental health professionals from WHP completed the participants’ consents, screening and baseline assessments. In the original plan, participants were assessed by personnel blinded to subject group assignment at three and six month time-points. Unexpected personnel and budget constraints required that the un-blinded study coordinator and un-blinded assistant carry out these assessments. The study coordinator and study assistant each conducted approximately half of the assessments. They worked independently and did not assess the same group of participants at each time-point.

### Treatments

#### Treatment as usual (TAU)

TAU included continuation of SSRIs, SNRIs, benzodiazepines and crisis counseling services. Those receiving medication met weekly with psychiatrists for medication management and had access to mental health professionals for interim crisis care. For those not on medication, TAU consisted of mental health crisis services, as needed.

### IPT + TAU

*IPT Adaptation.* Traditional IPT was modified slightly to address trauma-related mental disorders of the local population. IPT focuses on one of four areas, depending on the etiology of the patient’s distress –interpersonal disputes, role transitions, grief/loss or interpersonal sensitivity/deficit [17]. For the purposes of this study, the last category was eliminated, as it relates to lifelong patterns of relational deficits often driven by character traits, whereas the focus of this study was on testing IPT for trauma-related mental disorders.

IPT was delivered in one hour weekly individual sessions for 12 weeks. IPT participants also received TAU. For participants taking medication, IPT and medication management were delivered by the same clinician in the same weekly session.

Prospective study therapists were recruited through our collaborators and intensively trained in IPT for two weeks in June of 2011 by S Meffert. Those who completed the course and successfully modeled a beginning, middle and end session of IPT were invited to join the study as therapists—six women and four men. Five were psychologists working at Shifang Counseling Center, four were psychiatrists working at Shifang People’s Hospital, and one was a teacher experienced with processing emotional trauma secondary to the earthquake. Therapists were assigned one practice IPT case and study cases were added to caseloads as practice IPT sessions were successfully accomplished. IPT was supervised by an onsite psychologist-study coordinator (RF Jiang) or remotely by a psychologist located in San Francisco and originally from China (HQ Tong). Both were experienced with IPT delivery and spoke weekly with S Meffert to monitor therapists’ application of IPT and address any questions.

### IPT Adherence

Treatment fidelity was assessed for each session by the therapist supervisor. Supervisors rated therapists’ adherence to IPT protocol using a ten-point scale assessing overall quality of the session (three items) and quality of key components for each of four phases (2–5 items), and two reverse coded items for off-protocol treatments, such as CBT.

All primary and secondary measures were administered by research psychologists from WHP at each time-point (baseline, three and six months).

### Primary outcomes

Primary outcomes were diagnosis of PTSD on the CAPS and/or diagnosis of MDD on the SCID, both of which are widely used in China [4,18,19]. We are not aware of psychometric assessments (reliability or validity) focusing specifically on the use of the SCID/CAPS with a Chinese population.

### Secondary outcomes

A standard process of forward/backward translation with resolution of discrepancies was used [20-22]. Two mental health professionals from WHP, bilingual in English and the local Mandarian dialect and experienced with emotional distress in the local population, separately translated English versions into local Mandarian. Discrepancies were resolved through discussion and consensus. Two additional personnel then separately back-translated the measures from Mandarian into English, compared and resolved discrepancies. The Cronbach alphas observed in this sample were calculated using Proc Corr in SAS v9.3.

*Beck Depression Inventory (BDI-II)* is a widely used 21-item depression symptom measure [23]. It has shown strong convergent validity with other measures of depression and has a Cronbach’s alpha of .91 for outpatient populations [24]. The psychometric properties of the Chinese BDI have been tested with other populations in China, demonstrating strong reliability with Cronbach’s alpha of .85, and strong construct validity with the exception of the item addressing loss of libido [25]. Cronbach’s alpha in this sample was .92.

*Generalized Self-Efficacy (GSE) is a* 10-item psychometric scale designed to assess optimistic self-beliefs [26]. Across different language versions, the GSE Cronbach’s alpha ranges from .75 to .91 and has convergent validity with other social cognitive variables [27]. Cronbach’s alpha in this sample was .86.

*State Trait Anger (STAXI) is* a widely used measure of state and trait anger consisting of 15 and 10 items, respectively. Cronbach’s alpha has been shown to be .93 and .86 for state and trait scales, respectively [28]. Construct (convergent) validity is supported by its high correlation with the Buss-Drukee and Cook-Medley scales. A Chinese version of the STAXI has been tested with two samples of Hong Kong populations, which showed supported construct validity and reliability [29]. The Cronbach’s alphas for this sample were .93 (STAXI-state) and .85 (STAXI-trait).

*Conflict Tactics Scale (CTS)* is a 10-item widely used scale for identifying intimate partner maltreatment. For the purposes of this study, we used the CTS subscales corresponding to IPV, including total couple violence, respondent’s victimization and perpetration. Cronbach’s alphas for the scales are .88, .83 and .82, respectively. Construct validity of the CTS is supported by correlation with other measures of aggression [30]. Cronbach’s alpha for this sample was .90.

*Social Adjustment Scale (SAS)* is a 54-item scale measuring role performance. The SAS has established construct and criterion validity and Cronbach’s alpha of .74 [31]. In this sample, the SAS social/leisure subscale had a Cronbach’s alpha of .82.

*Quality of Life Index (QLI)* is a widely used 24-item scale that measures well-being. The QLI has established construct and convergent validity with a Cronbach’s alpha of .93 for the overall scale and .87 for the health and functioning subscale [32]. In this sample, the overall scale and the health/functioning subscale had Cronbach’s alphas of .95 and .73, respectively.

### Statistical analysis

Data was described using standard summary statistics. The main analysis consisted of comparing the change from baseline to post-test (three months) between the two treatment conditions. The two primary outcome measures of PTSD and MDD diagnosis were analyzed by estimating and testing a statistical model of each dichotomous measure with a binomial distribution and logit link using Generalized Estimating Equations to account for clustering of participants by therapist (therapist effect) and the repeated assessments. An unstructured covariance matrix was specified. Models included terms for time (baseline and post-test), treatment condition, their interaction and the participant’s gender. Parallel models of the secondary scales were also tested using a continuous distribution in place of the binomial. For these tests of treatment effect, all available data was used to estimate and test the statistical models.

Given the limited sample size and the focus on end-of-treatment effects, the percent in each condition diagnosed with PTSD or MDD and their associated symptom levels were tested for significant change from the three-month post-test to the six-month follow-up within each of the conditions using McNemar’s test and repeated measures t-tests. All analyses were conducted under the intent-to-treat principle and all available data was used on testing. Analysis was conducted using SAS v9.3.

## Results

**Figure 1:**
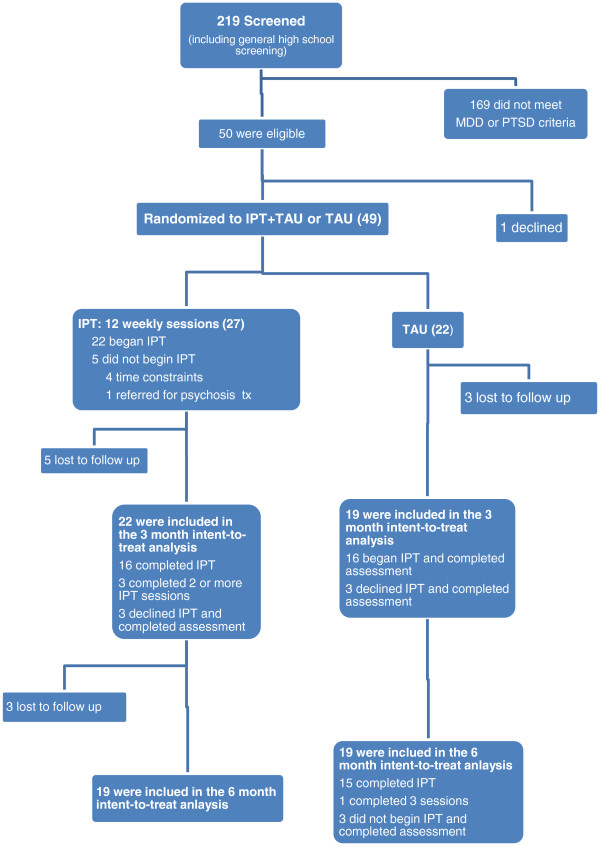
**Flow of participants through study.** IPT: Interpersonal Psychotherapy; MDD: Major Depressive Disorder; PTSD: Posttraumatic Stress Disorder; TAU: Treatment As Usual; Tx: Treatment.

A total of 219 participants were screened August-November 2011 (Figure 1). 207 of these were part of a general screening of all students attending a high school with heavy earthquake exposure, of which 38 met study criteria and 37 agreed to participate (18%). Twelve participants were referred from mental health clinics (Shifang Counseling Center and Shifang People’s Hospital) – all met study eligibility criteria and all agreed to participate. In total, of the 50 eligible study participants, 49 (98%) agreed to participate.All study participants completed baseline measures. Twenty-seven participants were assigned to IPT + TAU. Of the 22 individuals who began IPT, 19 completed two or more sessions (86%) and 16 completed all 12 sessions (73%). Five of the IPT + TAU group were lost to follow-up between baseline and the three-month assessment, including one who moved away from the area. Of the 22 participants assigned to TAU, 19 participants completed the three-month assessment. Sixteen of the TAU participants began IPT when it was offered at three months; 100% of these completed two or more sessions and 93% completed all 12 sessions (Figure 1). One participant assigned to a psychologist was identified as having symptoms of psychosis prior to starting IPT and was referred to a psychiatric inpatient unit for hospitalization and medication. None of the other participants in either group required crisis counseling, medication changes/initiation or psychiatric consultation during the study.

Baseline sample characteristics of the participants are shown in Table 1. 18.5% of the IPT + TAU group was taking SSRI/SNRI and benzodiazepine, 3.7% was taking only SSRI/SNRI (22% total taking medication). 9.1% of the TAU group was taking SSRI/SNRI and benzodiazepine and 9.1% for was taking only SSRI/SNRI (18% total taking medication). The majority of study participants were women and most identified the 2008 earthquake as their most stressful life event.

**Table 1:** Baseline characteristics of study sample

**Variable**	**IPT + TAU (n = 27)**	**TAU (n = 22)**
	**Mean/number**	**Mean/number**
	**(SD or%)**	**(SD or%)**
Age	24.79 (11.66)	36.05 (15.68)
Women	18 (62)	17 (77)
Medication (total)	6 (22)	4 (18)
SSRI/SNRI + BZ	5 (18.5)	2 (9.1)
SSRI/SNRI	1 (3.7)	2 (9.1)
**Most traumatic life event**		
Earthquake	21 (72)	20 (77)
Interpersonal	2 (7)	0
Other	4 (15)	2 (9)

BZ: benzodiazepine; IPT: Interpersonal Psychotherapy; SD: Standard Deviation; SNRI: Selective Serotonin and Norepinephrine Reuptake Inhibitor; SSRI: Selective Serotonin Reuptake Inhibitor; TAU: Treatment as Usual.

At baseline, 45.5% of the TAU and 66.7% of the IPT + TAU participants were positive for PTSD (Table 2). At the post-test, 42.1% in the TAU and 13.6% of the IPT + TAU condition had PTSD. GEE parameter estimates were −2.58 for time (p = .001), −1.52 for condition (p = .055) and 2.37 for time by condition (p = .018). At baseline, 59.1% of the TAU and 51.9% of the IPT + TAU participants were positive for MDD (Table 2). At the post-test, 57.9% in the TAU and 18.2% of the IPT + TAU condition were positive. GEE parameter estimates were −1.75 for time (p = .023), −1.99 for condition (p = .007) and 1.94 for time by condition (p = .056). Secondary outcome baseline scores were similar between groups (Table 3). For those participants in the IPT + TAU group who had MDD at baseline, 92% had remitted and no longer met criteria for MDD following treatment (three months). IPT + TAU participants with PTSD at baseline had a remission rate of 58% at three months. In comparison, at three months, TAU recipients had remission rates of 30% and 0% for MDD and PTSD, respectively. At the six-month follow up, the IPT + TAU group maintained treatment gains and had further reduction of PTSD and MDD diagnoses. Upon receipt of IPT, the TAU group demonstrated reductions in PTSD and MDD diagnoses similar to that observed in the IPT group. Neither therapist nor supervisor assignment had an effect on IPT outcomes.

**Table 2:** Primary outcomes: baseline and 3 months by group

**Variable**	**IPT + TAU (n = 27)**		**TAU (n = 22)**		**Chi-Square**
**Baseline**	**No.**	**%**	**No.**	**%**	
PTSD diagnosis (CAPS)	18	66.7	10	45.5	2.05
Depression diagnosis (SCID)	14	51.9	12	59.1	.04
**3 Months**	***Post-IPT (n = 22)***		***Post-TAU (n = 19)***		
PTSD diagnosis (CAPS)	3	13.6	8	42.1	4.21
Depression diagnosis (SCID)	4	18.2	11	57.9	6.93*

*p ≤ .01.

CAPS: Clinician Administered Posttraumatic Stress Disorder (PTSD) Scale; IPT: Interpersonal Psychotherapy; PTSD: Posttraumatic Stress Disorder; SCID: Structured Clinical Interview for DSM-IV; SD: Standard Deviation; SNRI: Selective Serotonin and Norepinephrine Reuptake Inhibitor; SSRI: Selective Serotonin Reuptake Inhibitor; TAU: Treatment as Usual.

**Table 3:** Secondary outcomes: baseline and 3 months by group

	**IPT + TAU (n = 29)**		**TAU (n = 22)**		
**Baseline**	**No. or mean**	**SD or %**	**No. or mean**	**SD or %**	
PTSD symptoms (CAPS)	39.41	15.38	45.05	11.06	
Depression symptoms (BDI)	20.7	11.6	21.8	12.7	
Anger (state)	23.83	8.61	26.38	11.34	
Anger (trait)	19.45	5.43	20.86	5.24	
Quality of life index(QLI)	17.12	5.07	14.07	3.73	
QLI Health and function subscale	15.93	5.90	13.03	3.82	
Social support (SAS)	2.93	.79	3.05	.67	
Social/leisure subscale					
Interpersonal conflict (CTS)	1.15	2.92	2.10	6.51	
Partner violence subscale					
Self-Efficacy	24	.90	24.18	.90	
	**IPT + TAU (n = 19)**		**TAU (n = 19)**		
**3 Months**	**No. or mean**	**SD or %**	**No. or mean**	**SD or %**	**Cohen d (row)**
PTSD symptoms (CAPS)	19.59	17.94	38.74	19.76	−1.01
Depression symptoms (BDI)	10.6	13.2	20.7	12.5	-.79
Anger (state)	17.8	5.7	25.58	10.78	-.90
Anger (trait)	17.00	4.77	20.37	5.28	-.67
Quality of life index(QLI)	19.90	6.07	15.07	4.62	.90
QLI Health and function subscale	19.40	6.98	14.07	4.60	.90
Social support (SAS)	2.47	.70	3.03	.61	-.85
Social/leisure subscale					
Interpersonal conflict (CTS)	.18	.50	1.74	5.77	-.38
Partner violence subscale					

BDI: Beck Depression Inventory; CAPS: Clinician Administered Posttraumatic Stress Disorder (PTSD) Scale; CTS: Conflict Tactics Scale; IPT: Interpersonal Psychotherapy; PTSD: Posttraumatic Stress Disorder; QLI: Quality of Life Index; SAS: Social Adjustment Scale; SD: Standard Deviation; TAU: Treatment as Usual.

**Figure 2:**
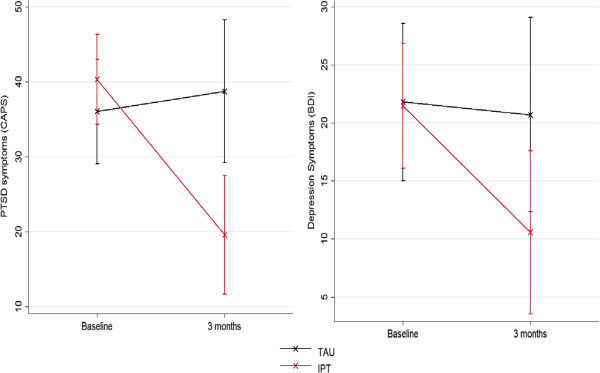
PTSD symptoms (CAPS*) and Depression symptoms (BDI*): IPT and TAU. *CAPS symptom scale is 0–600 and BDI symptom scale is 0–63; both are expanded here for visibility. BDI: Beck Depression Inventory; CAPS: Clinician Administered Posttraumatic Stress Disorder (PTSD) Scale; IPT: Interpersonal Psychotherapy; PTSD: Posttraumatic Stress Disorder; SD: Standard Deviation; TAU: Treatment as Usual. 95% confidence intervals shown.

Analysis of PTSD and depression symptoms (as opposed to diagnoses) also found a strong IPT effect (Figure 2). Cohen’s d effect sizes for PTSD symptoms on CAPS and depression symptoms on the BDI were 1.01 and .79, respectively (Table 3). Significant effects were found on all three models terms of time, condition and the interaction for CAPS symptoms and BDI symptoms. We conducted exploratory analyses of the effects of gender and medication use on IPT response. Gender had no effect on IPT response for PTSD or MDD diagnosis or symptoms. Use of antidepressant (SSRI or SNRI) or benzodiazepines did not predict diagnosis with PTSD or MDD, however, it was associated with higher PTSD and depression symptoms. IPT remained a significant predictor of diagnosis and symptom response even in the presence of antidepressant and benzodiazepine use.

Although the study was not designed to detect changes in anger and interpersonal violence, IPT had moderate-large effects on anger and violent victimization by an intimate partner (Table 3). The term for condition was significant for trait anger and just missed the cutoff for state anger (p = .053). IPT also had a large effect on increasing social adjustment on the SAS social/leisure subscale. The social/leisure subscale was significant for time, condition and interaction, as were the student and parental subscales when applicable to the participant. IPT had a large effect on increasing self-efficacy, quality of life total score and the health and function subscale. Significant effects were found on all three models terms of time, condition and the interaction for self-efficacy. The quality of life total score and health and function subscale showed significant change over time and condition.

Mean IPT adherence was 8.0 with a range of 7.3 to 9.7. We observed a graduated response for MDD such that greater adherence to IPT protocol was significantly and inversely associated with MDD diagnosis at the conclusion of treatment.

## Discussion

IPT delivered by local personnel was effective for reducing chronic PTSD and depression symptoms, as well as full diagnosis of PTSD and MDD among Sichuan earthquake survivors. IPT increased overall quality of life, social support and self-efficacy, while reducing anger and receipt of violent victimization. Treatment gains were maintained three months following completion of the intervention with further spontaneous improvement, although additional follow up is necessary to assess maintenance of treatment gains. Similar reductions in PTSD and depression were observed for TAU participants after they received IPT.

Although this study was not designed to detect the relational effects of IPT, we found lowered rates of intimate partner violence (IPV) toward IPT + TAU recipients, consistent with a psychotherapy trial of IPV survivors in the U.S. which found that reduction of PTSD and depression was associated with lower risk of future IPV [33]. Iverson and colleagues assessed the effect of CBT for depression and PTSD among female survivors of interpersonal violence on IPV in the six months following the study (*n* = 150). Reductions in PTSD and depression were associated with decreased risk of IPV in the six months following the study, controlling for recent IPV in a current relationship. Treatment response correlated with lower IPV levels at the six-month follow-up, such that individuals who experienced a greater reduction of PTSD and/or depression symptoms reported lower IPV levels at six-month follow-up compared with those who had poorer treatment response. Although the mechanism is not yet clear, Iverson and colleagues drew on PTSD literature, positing that numbing symptoms may put women at risk of IPV by impairing their ability to detect and respond to danger cues. Depression was theorized to affect victimization by decreasing cognitive and affective capacity, as well as self-worth. Clearly, neither the Iverson study nor the secondary findings presented here suggest that IPV is in any way brought on by the survivor, but rather that improving the mental health of an IPV survivor may provide them with better access to their full emotional and cognitive capacities in order to grapple with the threat of future IPV. Of course, mental health treatment of IPV survivors could never supplant strenuous efforts halt perpetration; rather such treatments might augment larger societal efforts to change gender norms and reduce IPV.

IPT + TAU participants who were parents reported improvement in the quantity and quality of interactions with children. Although the numbers are small, these findings together with observed improvements in social support are encouraging in regards to IPT’s potential contribution to social reconstruction in traumatized communities. In a post-disaster setting with highly disrupted social structures, it is possible that an interpersonally focused treatment such as IPT may both improve symptoms of depression and PTSD, and reconstitute social support, a central factor in recovery from traumatic stress [34-36]. However, it is important to note that this study took place two years after the earthquake and findings may not be generalizable to the immediate disaster response.

This study used a brief training period, followed by onsite or remote supervision. The success of remote supervision with minimal ground support is promising, given the need for global mental health care scale up.

In this study, the cost of one PTSD and/or MDD cure was approximately 200USD for therapist payment (total cures/therapist payments) and 177USD for therapist supervision (total cures/therapist supervisor payments)—the latter is expected to decrease as therapists gain experience. For comparison, cure of drug-sensitive TB is ~250USD and one year of HIV treatment in sub-Saharan Africa averages ~900USD [37-39].

### Limitations

The primary limitation of this study is its size and follow up. As a pilot study, the goal was to determine the efficacy of IPT for common mental health sequelae following the Sichuan earthquake. Larger size and longer follow up were limited by financial constraints.

A second limitation of this study is the lack of blinding to treatment status, which could introduce bias in favor of IPT efficacy. The possibility of bias may be mitigated by the fact that both symptom and diagnostic measures showed similar patterns of IPT response, and that spontaneous remissions were observed in the TAU and the IPT + TAU group in non-treatment phases.

Given the length of time between the earthquake and the start of this study, most participants had chronic psychopathology. It is encouraging that IPT was effective for chronic PTSD/MDD, but findings may not be generalizable to populations more recently affected.

The use of TAU, rather than a therapy control designed to meet with the same frequency and duration of IPT, limits the interpretation of the data in regards to the mechanism of change fostered by IPT.

Finally, given the size of the samples required to conduct psychometric analyses, the measures used in this study were not validated with the local Sichuan population. Although widespread use of CAPS and SCID in China [18,19,40] does not guarantee validity, the symptom measures, including the BDI and the PTSD Checklist (PCL) (latter not reported here), showed a pattern of results similar to those observed with diagnostic measures, suggesting convergent validity.

## Conclusions

This study contributes to the field of global mental health research by providing data on use of IPT in China and first use with survivors of natural disaster. This and other studies [7-9,15,41,42] now constitute a sizable body of research showing efficacy for culturally-adapted IPT and other evidence-based psychotherapies delivered in a feasible and effective manner by local personnel for common mental disorders in LMICs. Given the pressing need to find sustainable treatments to address the mental health care gap [43-45], the accumulation of this data is exciting. Larger studies with long-term follow up and active controls, as well as cost-effectiveness measures are next steps for global mental health intervention research with traumatized populations.

## Competing interests

The authors declare that they have no competing interests.

## Authors’ contributions

RJ supervised IPT, carried out data collection, compiled data for analysis and assisted with drafting the manuscript. HT supervised IPT, assisted with data collection and assisted with drafting the manuscript. KD conceptualized and performed the data analysis. TN and QS assisted with study design and execution. SM conceived of the study, participated in its design, execution, data analysis and drafted the manuscript. All authors read and approved the final manuscript.
